# A continuous intravenous insulin infusion protocol to manage high-dose methylprednisolone-induced hyperglycemia in patients with severe COVID-19

**DOI:** 10.1186/s40842-022-00141-2

**Published:** 2022-04-27

**Authors:** Yoshihito Takahashi, Hiroshi Matsuura, Hisaya Domi, Hitoshi Yamamura

**Affiliations:** Osaka Prefectural Nakakawachi Emergency and Critical Care Center, 3-4-13 Nishiiwata, Higashiosaka, Osaka 578-0947 Japan

**Keywords:** Blood glucose, Methylprednisolone, Protocol

## Abstract

**Background:**

Many patients with severe COVID-19 have impaired glucose tolerance, and steroid therapy is a standard treatment. Thus, good glycemic control is important and correlates with better patient outcomes. We began using a continuous intravenous insulin infusion protocol for glycemic control whose infusion rate changes based on the currently measured value and previous value. This study aimed to evaluate this protocol for COVID-19 patients requiring mechanical ventilation.

**Methods:**

This single-center, retrospective, case control study was conducted on all adult patients who required mechanical ventilation for severe COVID-19 pneumonia admitted to our critical care center from April 1, 2020 through June 20, 2021. Blood glucose levels were measured in all patients every 4 h after admission. We started using the insulin infusion protocol from August 1, 2020. Patients before starting the protocol comprised the non-protocol group and those after starting the protocol comprised the protocol group. Blood glucose levels and hypo- or hyperglycemia events were compared between groups. We also surveyed ICU nurses about their experience using the protocol.

**Results:**

During the study period, 173 patients with COVID-19 were admitted. After 15 patients were excluded for several reasons, the study included 158 patients: non-protocol group (*n* = 14) and protocol group (*n* = 144). In the initial phase (days 1–2), blood glucose levels of the protocol group were higher compared with the non-protocol group, and as the number of measurements increased, blood glucose levels were gradually brought under control within the target range in the protocol group. Almost no hypoglycemic events (blood glucose < 80 mg/dL) were detected in either group. The rate of hyperglycemia (blood glucose > 300 mg/dL) was about 5–10% in the initial phase in the protocol group and about 10–15% in the early phase (days 3–4) in the non-protocol group. The questionnaire survey revealed that 80% of ICU nurses responded favorably.

**Conclusions:**

This insulin protocol gradually brought the blood glucose level within target levels in severe COVID-19 patients treated with high-dose steroid. Some hyperglycemia events were detected despite patients being under the protocol in the initial phase, and thus, minor modifications of the protocol might be required in the initial phase.

## Background

The novel coronavirus disease 2019 (COVID-19) has emerged as a global pandemic and has claimed many lives to date [1, 2]. Diabetes mellitus (DM) is one of the important distinctive comorbidities associated with severity, acute respiratory distress syndrome, and increased mortality in COVID-19 patients [3]. Especially, patients with type 2 diabetes have shown higher risk for developing more a severe case and have suffered higher risk of mortality [4]. In type 2 diabetes patients with COVID-19, improved glycemic control correlates with better outcomes [5], and maintaining good glycemic control can boost the innate immune system and help prevent grave consequences. Thus, appropriate glucose control is an important therapeutic target in the care of these patients in the intensive care unit (ICU).

Many patients with severe COVID-19 have impaired glucose tolerance [5, 6], and steroid therapy is one of the standard treatments [7], thus making control of blood glucose important in the ICU. A glycemic control protocol for COVID-19 has been reported as a sliding scale for subcutaneous injection of insulin [8], and continuous insulin infusion adjustments were also reported in some studies of small numbers of patients undergoing continuous glucose monitoring [9, 10]. Continuous intravenous insulin infusion is the best method for achieving glycemic targets in the critically ill patient [11], and several infusion protocols for ICU patients that included computer-guided management were reported previously [12–17]. The insulin doses in the previously published protocols are very high compared with usual dose administered in our ICU, and we think there would be a potential risk of hypoglycemia. Therefore, these protocols may not be appropriate for the Japanese physique, and the protocol for severe COVID-19 patients, of whom many have impaired glucose tolerance and all receive steroid therapy, has not been investigated adequately.

**Fig. 1:**
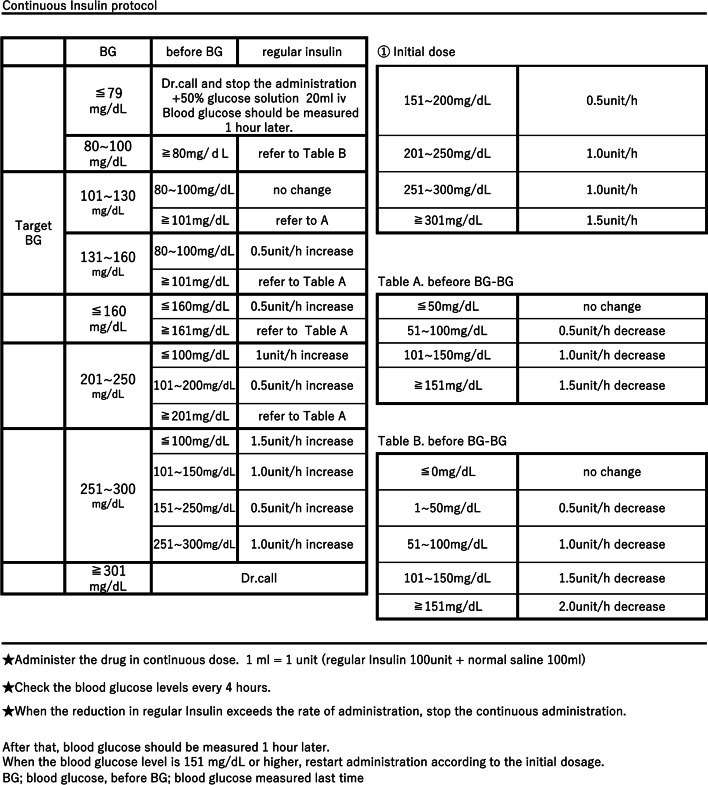
The continuous intravenous insulin infusion protocol

Since the beginning of the pandemic, we have accepted only severe COVID-19 patients who required mechanical ventilation in our critical care center. We have faced difficulty in controlling blood glucose levels, and adjustment of insulin infusion rates has increased the workload of the ICU staff. Thus, we introduced a continuous intravenous insulin infusion protocol for glycemic control in which the infusion rate was changed based on the currently measured value and the previous value (Fig. 1). We had already tried this protocol in the ICU in patients with other diseases and confirmed that the protocol was working. During the initial non-protocol period, there were many instances requiring a “Dr. call”, i.e., a call to an ICU physician regarding modification of patient treatment, which hindered the other duties of the ICU staff and thus motivated us to introduce this protocol. Blood glucose levels were evaluated by comparing them before and after we began use of the protocol.

The aim of this study was to investigate how the control of blood glucose levels in COVID-19 patients requiring mechanical ventilation was improved by using the protocol.

## Methods

### Study design

**Fig. 2:**
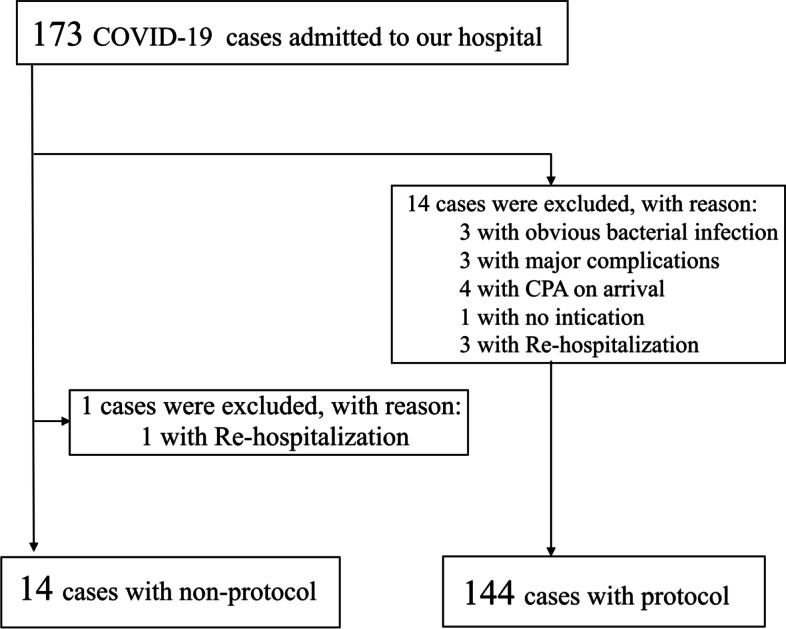
Patient flow diagram. CPA, cardiopulmonary arrest

This was a single-center, retrospective, case control study conducted on all adult patients admitted to our critical care center, including those transferred from other hospitals, from April 1, 2020 through June 20, 2021. All patients 18 years of age or older who required mechanical ventilation for severe COVID-19 pneumonia were included in the study. The criterion for intubation due to respiratory failure was the inability of the patient not to maintain a peripheral arterial oxygen saturation of 92–94% on oxygen inhalation of 5–10 L/min by mask. The non-intubated patients with a positive COVID-19 polymerase chain reaction test and those with obvious bacterial infection, major complications, cardiopulmonary arrest on arrival, or re-hospitalization due to the need for mechanical ventilation were excluded (Fig. 2). We started the insulin infusion protocol from August 1, 2020; before then, if the blood glucose level deviated from the target value, ICU physicians received a call from the ICU staff for modification of treatment.

We applied an opt-out method on the hospital’s web site to obtain patient consent. This study followed the principles of the Declaration of Helsinki and was approved by the Institutional Review Board for Clinical Research of the hospital (approval no.: 02–0606-A).

### Treatment protocol

We administered favipiravir or remdesivir in accordance with the patient’s previous hospital use or started favipiravir from admission during April 1, 2020 through April 21, 2021. From April 22, 2021, we administered remdesivir via intravenous drip infusion (200 mg on day 1, 100 mg from day 2). We also administered intravenous steroid to all COVID-19 pneumonia patients admitted to our ICU using the following treatment protocol: methylprednisolone (1000 mg for 3 days followed by 125 mg for 3 days and 40 mg for 3 days). In addition, we administered low molecular weight heparin (2000 IU every 12 h) or unfractionated heparin (10,000–24,000 IU/day). Heparin was administered after mechanical ventilation was started, and dexmedetomidine was started for sedation in all patients.

### The intravenous insulin infusion protocol for glycemic control

The blood glucose levels of all patients were measured every 4 h after their admission. Subcutaneous insulin was not administered in the acute phase in this study. Intravenous insulin was administered in accordance with the protocol (Fig. 1). Briefly, continuous doses (1 mL = 1 unit: regular insulin 100 u + normal saline 100 mL) were administered, and the protocol focused on the currently measured blood glucose level and that measured 4 h before. We set the target blood glucose level at 101–160 mg/dL in this protocol. When blood glucose levels of ≤ 89 or ≥ 301 mg/dL were measured, defined as the ‘‘Dr. call’’ values, the ICU nurses contacted ICU physicians to request modification of the insulin dose. When the reduction of regular insulin exceeded the rate of administration, continuous administration was discontinued. After discontinuation, the blood glucose level was measured 1 h later. When the blood glucose level rose to ≥ 151 mg/dL, administration was resumed according to the initial dosage in the protocol.

In the control (non-protocol) group, the patients were administered continuous intravenous insulin, and the nurses contacted the ICU physicians to modify an insulin dose according to the ‘‘Dr. call’’ values. Thus, blood glucose levels of the patients were dependent on the physician in charge that day.

### Data collection

Patients were followed up until hospital discharge or death. Patient information was collected from the medical record, which included demographic characteristics, pre-existing comorbidities, laboratory tests including HbA1c, severity scores, and therapeutic interventions in our hospital or the previous hospital. Severity was evaluated by Acute Physiology and Chronic Health Evaluation (APACHE) II score. Clinical follow-up data were collected up to June 20, 2021.

### Measurement of blood glucose levels

Blood glucose levels were measured using a blood glucose monitoring device (Pocket Chem™ BG PG-7320, Arkray Inc., Kyoto, Japan), or adopt the results of blood gas analysis. In this study, we measured blood glucose levels every 4 h to avoid contact with the patients as much as possible after their admission to the ICU.

### Questionnaire for ICU nurses

We conducted an anonymous questionnaire survey of the ICU nurses about their experience in using the insulin infusion protocol. The single question was “How about the insulin protocol?” The allowable responses were as follows: very good, good, bad, and very bad. We also asked the nurses to provide positive and negative free-style comments regarding the protocol.

### Statistical analysis

Patient age and other demographic data are expressed as median ± interquartile range (IQR) or counts and percentages. Laboratory data are expressed as median with IQR. The Wilcoxon signed-rank test and Fisher’s exact test were used to compare the COVID-19 patients’ data between the protocol group and non-protocol group. The serial changes of blood glucose levels in both groups are shown by mean ± standard deviation (SD). A *p* value < 0.05 was considered to be statistically significant. Statistical analyses were conducted with JMP Pro 16.0 for Windows (SAS Institute Inc., Cary, NC, USA).

## Results

### Patient characteristics

During the study period, 173 patients with COVID-19 were admitted to our hospital. We excluded patients for the following reasons: patients with obvious bacterial infection who did not receive high-dose corticosteroids and were intubated because of sepsis; patients with major complications in whom the reason for intubation was not COVID-19 pneumonia; all patients who were in cardiopulmonary arrest on arrival died at emergency room; and re-hospitalized patients treated with a different strategy. We thus excluded one patient in the non-protocol group because of re-hospitalization and 14 patients in protocol group, and finally, 158 patients were included in this study. The number of patients in the non-protocol group was 14 and that in the protocol group was 144 (Fig. 2). The patients’ clinical characteristics are shown in Table 1. No significant differences were detected between the non-protocol and protocol groups except for APACHE II score.

**Table 1:** Characteristics of the patients with COVID-19

	Total		non-protocol		protocol		p
	(*N* = 158)		(*N* = 14)		(*N* = 144)		
Age,median (IQR)	68.5	(56–74.3)	60	(50–73)	68	(56–74.8)	0.263
Sex, n (%)							
Male	107	(68)	10	(71)	97	(67)	
Female	51	(32)	4	(29)	47	(33)	0.756
BMI (kg/m2)(IQR)	25	(22.8–259)	24.1	(20.6–28.2)	25.2	(22.9–27.9)	0.345
P/F ratio after intubation (IQR)	203	(164–259)	213	(182.5–267)	198	(162.5–259.5)	0.343
APACHE ΙΙ score (IQR)	12	(9–14)	9	(5–13)	12	(9–15)	0.019
pre-existing diabetes, n (%)	44	(27.8)	1	(7)	43	(29.9)	0.07
HbA1c, median (IQR)	6.2	(5.8–7.2)	6.6	(6.0–7.8)	6.2	(5.8–7.2)	0.233
Intubation, n (%)	158	(100)	14	(100)	144	(100)	-
Anti-viral drug							
before admission							
Favipiravir, n(%)	51	(32)	0	(0)	51	(35)	-
after admission							
Favipiravir, n(%)	86	(54)	14	(100)	72	(50)	-
Remdesivir, n(%)	9	(6)	0	(0)	9	(6)	-
Steroid treatment							
before admission							
Dexamethasone, n (%)	57	(36)	0	(0)	57	(40)	-
Methylprednisolone, n (%)	34	(22)	0	(0)	34	(24)	-
after admission							
Methylprednisolone, n (%)	155	(98)	14	(100)	141	(98)	
ECMO, n (%)	2	(1)	1	(7)	1	(1)	
Mortality, n (%)	20	(13)	1	(7)	19	(13)	0.516

### Blood glucose levels after admission in both groups

**Fig. 3:**
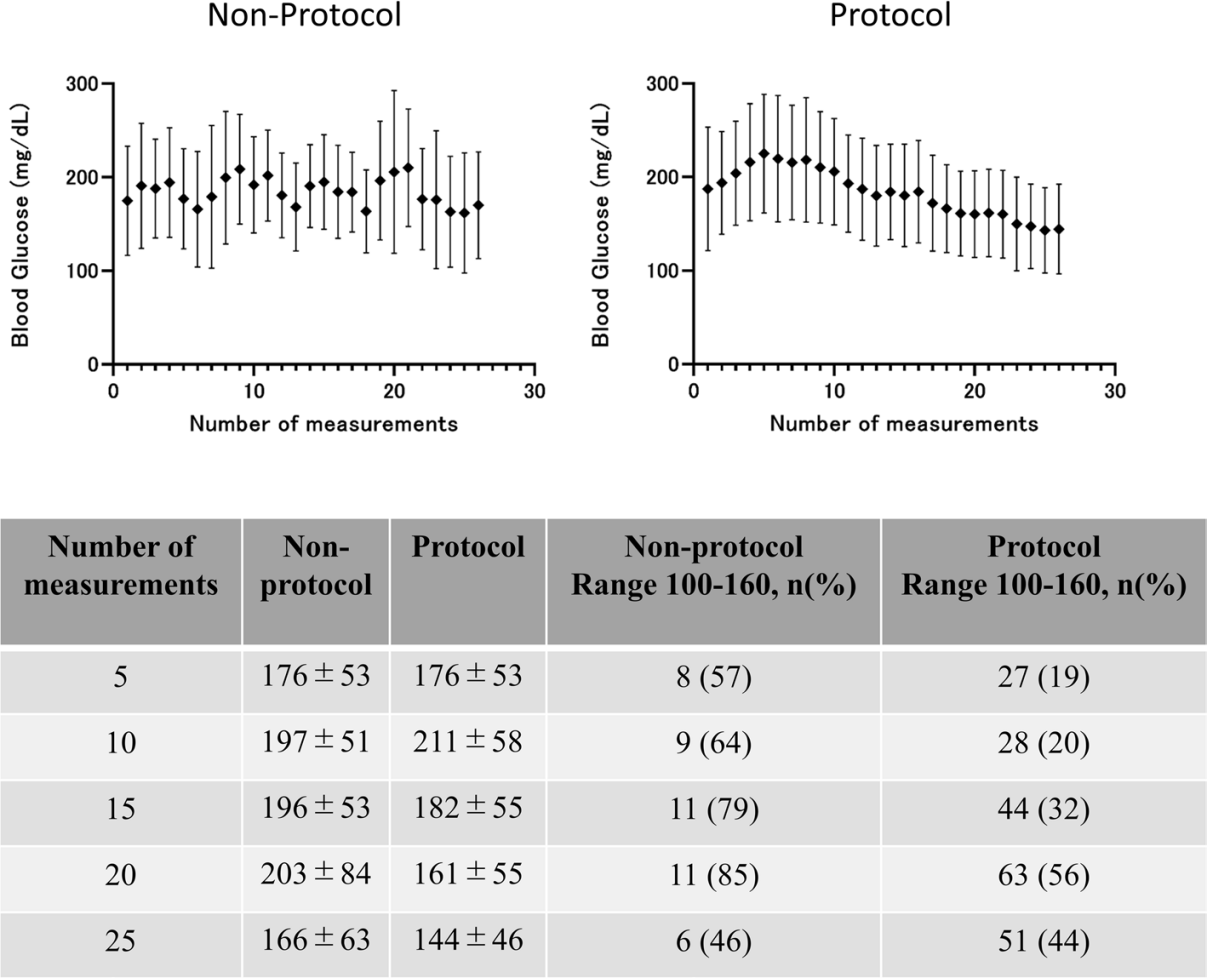
Serial changes of blood glucose levels in the protocol and non-protocol groups. Data are shown as mean ± SD

**Fig. 4:**
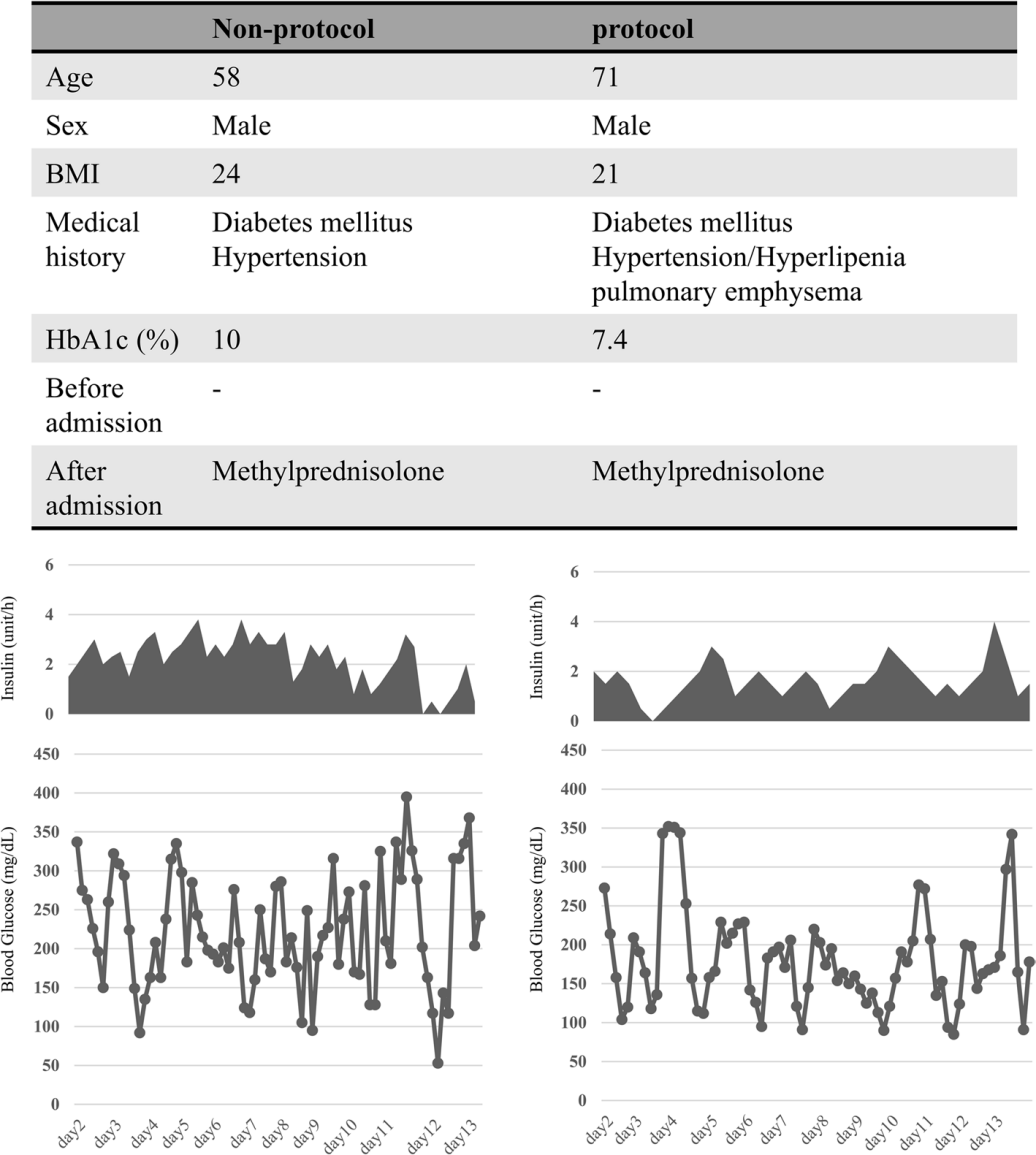
The clincial course of glucose and insulin levels in an example patient from each group

The blood glucose levels in the non-protocol and protocol groups every 4 h after admission are shown in Fig. 3. In the initial phase (days 1–2), blood glucose levels of the protocol group were higher compared with those of the non-protocol group. As the number of measurements increased, the blood glucose levels were gradually brought under control within the target range in the protocol group (Fig. 3). Average glucose values at various time points and percentages of readings were in the proposed target range of 100–160 mg/dL in each group as shown in Fig. 3. We also show the serial changes of glucose and insulin levels in an example patient from each group (Fig. 4).

### Evaluation of hypo- or hyperglycemia events

**Fig. 5:**
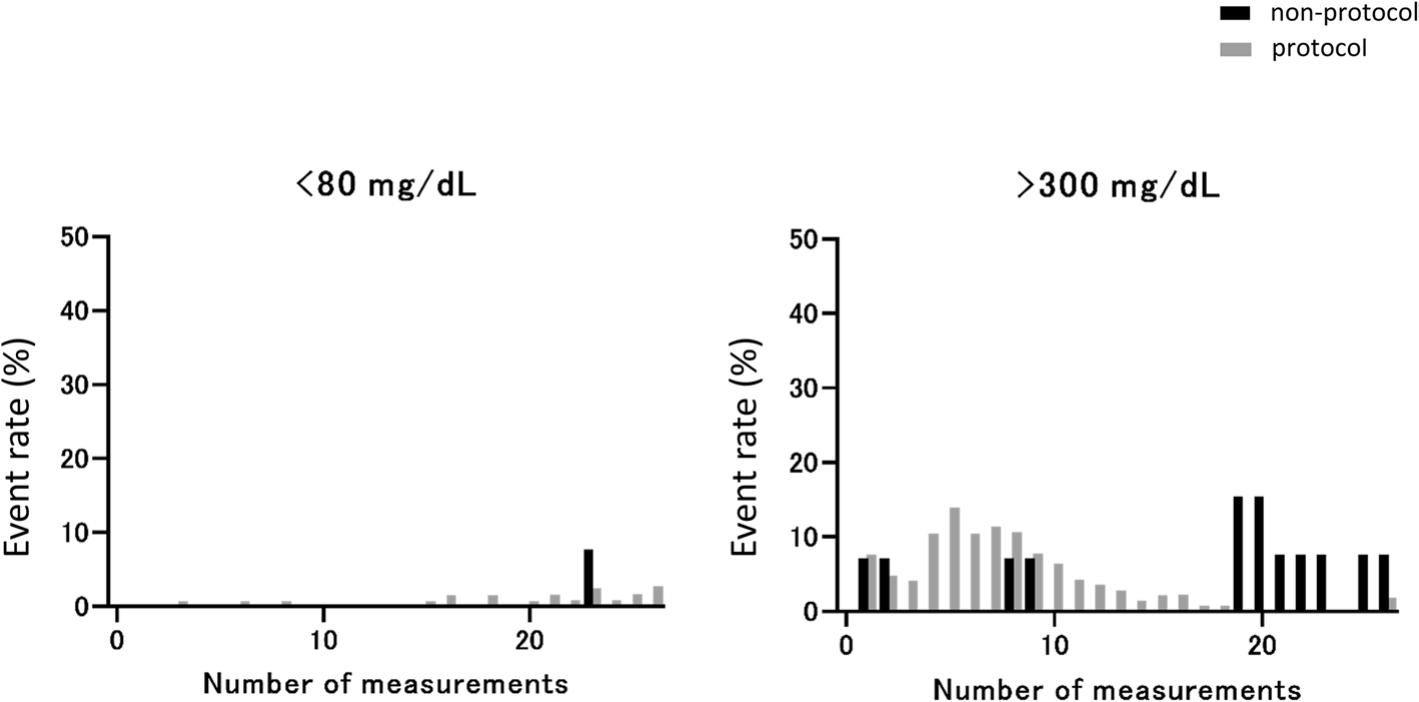
The hypo- or hyperglycemia event rates for blood glucose levels at < 80 mg/dL and > 300 mg/dL in both groups

We evaluated the hypo- or hyperglycemia event rates that required a “Dr. call” for treatment modification. Almost no hypoglycemia events (blood glucose < 80 mg/dL) were detected. The rate of hyperglycemia (blood glucose > 300 mg/dL) was about 5–10% in the initial phase in the protocol group and about 10–15% in the early phase (days 3–4) in the non-protocol group (Fig. 5).

### Questionnaire for ICU nurses

All 24 ICU nurses who used the protocol (100% response rate) responded anonymously to the questionnaire survey about the experience in using the insulin infusion protocol. The results revealed that 80% of the ICU nurses responded favorably: 20% thought it was very good, 60% good, and 20% thought it was bad.

## Discussion

In this study, we evaluated our intravenous insulin infusion protocol for glycemic control in severe COVID-19 patients treated with high-dose methylprednisolone therapy. The “cytokine storm” resulting from severe COVID-19 infection is associated with significant insulin resistance and reduced insulin production from pancreatic cells [18]. This dual pathology can precipitate severe hyperglycemia in people with diabetes and even in people without diabetes [18]. Our protocol was useful for patients whose glycemic control is difficult in the ICU.

A target blood glucose concentration of 144 to 180 mg/dL (8.0 to 10.0 mmol/L) is likely to reduce the risk of hypoglycemia in critically ill patients [19], and glucose concentrations above 180 mg/dL (10.0 mmol/L) have been linked to increased mortality in people with COVID-19 [20]. Clinically significant hypoglycemia has been defined as < 54 mg/dL (3.0 mmol/L), whereas a glucose alert value is defined as ≤ 70 mg/dL (3.9 mmol/L) [21]. The protocol set the target blood glucose levels at 101–160 mg/dL, and there were almost no instances of significant hypoglycemia. The blood glucose levels were gradually brought under control within the target glucose range as the number of measurements increased in the protocol group. However, in the initial phase, some hyperglycemic events were detected despite the patients being under the protocol. High-dose methylprednisolone therapy is used in our treatment protocol, and 1000 mg/day was administered for the first 3 days after admission. This is one of the main reasons for hyperglycemia, and thus, although the protocol may not be simple, minor modification of the protocol might be required in the initial phase.

In the questionnaire survey of the ICU nurses about their experience in using the insulin infusion protocol, 80% of the staff thought it was very good or good. One of main positive comments was that “nurses do not need Dr. call many times for adjusting blood glucose.” The other 20% responded negatively. The negative comments were as follows: “the protocol is complicated and there were some incidents” and “many nurse experienced large fluctuations in blood glucose even during using the protocol.” Further assessment and improvement of the protocol will be necessary in the future.

Our study has some limitations. First, this was a single-center, case–control study, and thus we could not assess the usefulness of the protocol in other ICUs. Second, the number of patients in the non-protocol group as the control was very small, and the event rates of hypo- or hyperglycemia in that group are uncertain. Third, although many studies have focused on the association between the hyperglycemic state and mortality, our study did not specifically assess a correlation between protocol use and patient prognosis. Finally, we think it is necessary to control the initial and subsequent insulin doses according to each patient’s background factors. To simplify the protocol, steroid pulse therapy with methylprednisolone at our center is administered according to the treatment protocol regardless of patient background factors. In a future study, we will need to modify parts of the protocol to better manage those patients in whom blood glucose control is difficult.

## Conclusions

In the present study, our continuous intravenous insulin infusion protocol gradually brought the blood glucose level within the target level and showed no inferiority compared to the non-protocol method. The protocol made it easy for ICU staff to obtain physician orders to control blood glucose levels. Although some aspects of the protocol still require improvement, we believe that the protocol is useful in patients with severe COVID-19 treated with high-dose steroids. Further studies are needed to better adjust the blood glucose levels in the initial phase of treatment after admission.

## Data Availability

The datasets used and/or analyzed during the current study are available from the corresponding author on reasonable request.
